# COMMUNITY-BASED IMPLEMENTATION OF A CAREGIVER EDUCATION PROGRAM, REACH-TX: RACIAL-ETHNIC GROUP COMPARISON

**DOI:** 10.1093/geroni/igz038.1650

**Published:** 2019-11-08

**Authors:** Jinmyoung Cho, Donald R Smith, Alan B Stevens

**Affiliations:** 1 Baylor Scott & White Health, Temple, Texas, United States; 2 Area Agency on Aging/Community Investment United Way of Tarrant County, Fort Worth, Texas, United States

## Abstract

We present the effect of racial/ethnic group difference on the impact of REACH TX on measures of quality of life as implemented by the Alzheimer’s Association North Central Texas Chapter. Five dimensions of quality of life (burden, depression, social support, self-care, and problem behaviors) were assessed at baseline and 6-month follow-up among three racial/ethnic groups of caregivers (White: 1,050; African American: 269; Hispanic: 176). Generalized estimating equations (GEEs) were used to assess racial/ethnic differences in the changes of quality of life after adjusting covariates. Significant interaction effects between racial/ethnic group and time (from baseline to follow-up) were found in burden, depression, and social support. White and Hispanic caregivers showed significant improvements, while the improvement among African American Caregivers was not statistically significant. The disparity in outcomes among diverse racial/ethnic groups in the program suggests the REACH TX intervention would benefit from tailoring interventions for African American caregivers.

